# Low‐Cost Intrinsic Flame‐Retardant Bio‐Based High Performance Polyurethane and its Application in Triboelectric Nanogenerators

**DOI:** 10.1002/advs.202412258

**Published:** 2024-12-30

**Authors:** Xiaoyu Zhang, Xixian Yan, Fanglei Zeng, Hao Zhang, Peiyao Li, Haiyang Zhang, Ning Li, Qingbao Guan, Zhengwei You

**Affiliations:** ^1^ Jiangsu Collaborative Innovation Center for Photovoltaic Science and Engineering Jiangsu Province Cultivation Base for State Key Laboratory of Photovoltaic Science and Technology Jiangsu Province Key Laboratory of Environmentally Friendly Polymer Materials School of Materials Science and Engineering Changzhou University Changzhou 213164 P. R. China; ^2^ State Key Laboratory for Modification of Chemical Fibers and Polymer Materials College of Materials Science and Engineering Institute of Functional Materials Research Base of Textile Materials for Flexible Electronics and Biomedical Applications (China Textile Engineering Society) Shanghai Key Laboratory of Lightweight Composite Shanghai Engineering Research Center of Nano Biomaterials and Regenerative Medicine Donghua University Shanghai 201620 P. R. China

**Keywords:** bio‐based materials, flame‐retardant materials, polyurethane, thermal runaway warning, triboelectric nanogenerators

## Abstract

Flammability is a significant challenge in polymer‐based electronics. In this regard, triboelectric nanogenerators (TENGs) have enabled a safe means for harvesting mechanical energy for conversion into electrical energy. However, most existing polymers used for TENGs are sourced from petroleum‐based raw materials and are highly flammable, which can further accelerate the spread of fire and harm the ecological environment. In addition, the existing intrinsic flame‐retardant TENGs are not elastic at room temperature, which may potentially damage the flexible equipment and harm firefighters. This study presents an intrinsic flame‐retardant bio‐based elastic phytic acid polyurethane (PUPA) synthesized using a simple and efficient one‐pot polycondensation. The cross‐linked structure and polar phosphorus‐containing segments of PUPA are fabricated into PUPA‐TENG, demonstrating a superior elasticity (elongation up to 660%), flame retardancy (UL94 V‐0), impact resistance (34.71 MJ m^−3^), and dielectric constant (*D*_k_ = 9.57). Consequently, this study provides a simple strategy for tailoring TENGs toward environmentally friendly and secure power generators and electronics, which can effectively reduce fire hazards and potentially be applied to other fire‐risk fields such as personal protection, firefighting, and new energy.

## Graphical abstract

This study presents an intrinsic flame‐retardant bio‐based elastic phytic acid polyurethane (PUPA), demonstrating a superior elasticity (elongation up to 660%), flame retardancy (UL94 V‐0), impact resistance (34.71 MJ m^−3^), and dielectric constant (Dk = 9.57). A simple strategy is provided for tailoring TENGs toward environmentally friendly and secure power generators and electronics, which can effectively reduce fire hazards.

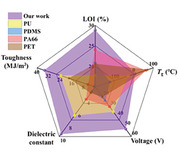

## 1 Introduction

Energy security is becoming an important global strategic issue.^[^ ^1^ ^]^ Most electronic devices currently require an external power supply,^[^ ^2^ ^]^ which is a potential source of fire hazard,^[^ ^3^ ^]^ furthermore, the large volume occupies a significant amount of space. Conversely, triboelectric nanogenerators (TENGs) have been demonstrated to be promising for renewable energy conversion based on triboelectric and electrostatic effects, as can be manufactured as a safer, more compact, and flexible device.^[^ ^4^ ^]^ By collecting a significant amount of available mechanical energy from the environment and converting it into electricity,^[^ ^5^ ^]^ TENGs can respond to mechanical signals and provide clean power for intelligent electronics.^[^ ^6^ ^]^ However, most existing polymers used to prepare TENGs are flammable,^[^ ^7^ ^]^ which may further accelerate the spread of fire.^[^ ^8^ ^]^

Incorporating flame‐retardants into polymers used to be the most popular means of improving the fire resistance of polymers.^[^ ^9^ ^]^ However, this method has led to several shortcomings, including a complex operation, migration of small molecular additives, deterioration of intrinsic properties of the matrix, and release of harmful combustion by‐products.^[^ ^10^ ^]^ For example, Chen et al. prepared an anti‐impact and flame‐retardant elastomer (AFE) for TENG by combining urea and carbon nanotubes with a shear‐stiffening elastomer. The AFE exhibited a limiting oxygen index of up to 30.5%; however, its preparation was complex, and toxic gases such as ammonia may be released during combustion.^[^ ^11^ ^]^ Therefore, developing intrinsic flame‐retardant polymers for new‐generation TENGs is crucial. We recently synthesized an intrinsic flame‐retardant all‐aromatic liquid crystalline poly(aryl ether ester) (LCP_AEE_) via a simple and efficient one‐pot melt polycondensation,^[^ ^12^ ^]^ however, the glass transition temperature (*T*_g_) of LCP_AEE_ was significantly high (115 °C), and thus, it was not elastic at room temperature (25 °C).^[^ ^5c^ ^]^ Rigid TENGs may be potentially harmful to flexible equipment and firefighters; furthermore, rigid materials cannot adapt to complex shapes and environments, thus severely limiting their application. In addition, LCP_AEE_ was prepared from petroleum‐based raw materials, which is a remaining concern considering the sustainable development of the ecological environment. In summary, developing intrinsic flame‐retardant bio‐based triboelectric polymers that are elastic (*T*_g_ < 20 °C) at room temperature is critical for TENG; however, this has not been achieved thus far.

**Figure 1:**
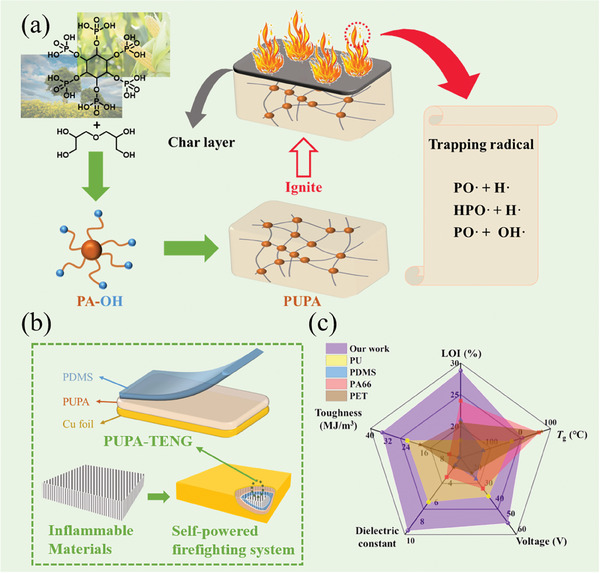
Illustration of self‐powered firefighting system based on TENG using newly designed PUPA, and its comparison with other existing triboelectric polymers. a) Preparation route and flame‐retardant mechanism of bio‐based PUPA. b) Design of PUPA–TENG. c) Comparison of representative triboelectric polymers and PUPA600‐1.5 properties prepared in this study.

In this study, phytic acid polyol (PA–OH) was first designed and prepared using phytic acid (PA) and diglycerol (DG) as two low‐cost bio‐based raw materials; subsequently, the intrinsic flame‐retardant bio‐based polyurethane elastic triboelectric material (PUPA) was synthesized via a stepwise polymerization method, as shown in **Figures** **1**a and S1 (Supporting Information). Compared to the traditional flame‐retardant polymer utilizing additives, the inherent flame retardancy and self‐extinguishing properties of PUPA enable it to be fabricated into a fire‐resistant triboelectric nanogenerator (PUPA‐TENG) without any modification or additives (Figure 1b). The resulting PUPA‐TENG exhibited excellent toughness and electrical output performance. Therefore, PUPA–TENG has significant potential as an easy‐to‐use, safe, and environmentally friendly power source, which can be applied to firefighting and personnel protection equipment in fire‐risk environments (Figure 1c).

## 2 Results and Discussion

Bio‐based Phytic acid polyol (PA–OH) and corresponding intrinsic flame‐retardant polyurethane elastomers (PUPAs) were designed and prepared, which have been verified by nuclear magnetic resonance spectroscopy, infrared spectroscopy, and energy dispersive spectrometer (EDS) tests. (Figure 1a; Figures S1–S3 and Tables S1–S2, Supporting Information)

**Figure 2:**
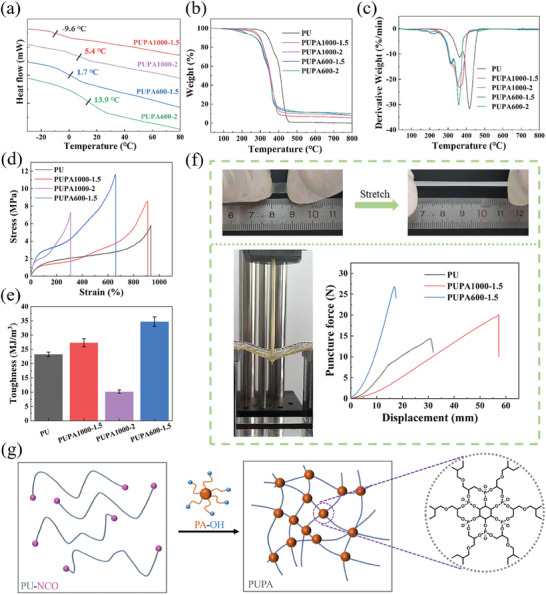
Thermal, morphological, and mechanical properties of synthesized PUPAs. a) DSC results (heating rate of 10 °C∙min^−1^ under nitrogen atmosphere). b) Thermogravimetric and c) derivative thermogravimetric analyses (heating rate of 20 °C∙min^−1^ under nitrogen atmosphere). d) Stress–strain curve. e) Fracture toughness. f) Elasticity and puncture resistance of PUPA600‐1.5 at room temperature. g) Toughening mechanism.

The *T*_g_ value of the PUPAs was lower than 20 °C, which was evaluated using differential scanning calorimetry (DSC), implying that they were soft and elastic at room temperature (**Figure** **2**a). A thermogravimetric analysis of the PUPAs is crucial to determine the thermal stability of the triboelectric‐layer materials. As shown in Figure 2b,c, the initial decomposition temperature (*T*_d5%_) of the ordinary PU reached 325 °C. As the content of PA–OH increased, the *T*_d5%_ value of the PUPAs demonstrated a downward trend; The derivative thermogravimetric analysis results indicate that as the PA–OH content increases, PUPA undergoes faster mass loss at lower temperatures. However, the char yield at 800 °C increased from 0% to 9.87% (Table S3, Supporting Information). This was owing to the preferential decomposition of the phosphorus‐containing segments in the PUPAs.^[^ ^13^ ^]^ Accordingly, this process provided PO· to capture the H· and OH· radicals, and promoted the formation of a carbon layer on the surface of the PUPAs, thereby effectively reducing the flame propagation rate and enhancing flame retardancy.^[^ ^14^ ^]^ These results indicate that PUPAs possess excellent thermal properties and can be used as promising candidates as elastic flame‐retardant triboelectric‐layer materials.

The mechanical properties of the PUPAs were investigated using a universal material testing machine. The stress–strain curves of the different samples are shown in Figure 2d; the fracture toughness of the different samples was calculated based on the area covered by the stress–strain curve (Figure 2e). The elongation at break of the ordinary PU was 934%; however, the fracture toughness was only 23.36 MJ m^−3^. PA–OH is a multifunctional rigid polyol structure, which can be introduced to strengthen PUs, thereby effectively improving the fracture toughness. However, the ultra‐high crosslinking density may restrict the movement of molecular chains,^[^ ^15^ ^]^ thereby deteriorating the fracture toughness of PUPA. Among the samples, the mechanical property of PUPA600‐2 was not reported owing to its high brittleness at room temperature, as demonstrated by the high gel content (85%) shown in Figure S5 (Supporting Information), and could not be clamped on a fixture for testing. Therefore, determining the appropriate amount of PA–OH to be added is crucial; in this regard, PUPA600‐1.5 was found to be the optimal formulation with a fracture toughness of 34.71 MJ m^−3^, which was 48% higher than that of the ordinary PU counterpart. Moreover, PUPA600‐1.5 exhibited excellent elasticity and puncture resistance in terms of the high fracture elongation (660%) and puncture force (27 N) at room temperature (Figure 2f and Figure S6, Supporting Information). The possible toughening mechanism is shown in Figure 2g. After introducing PA–OH, numerous rigid crosslinking sites are formed,^[^ ^16^ ^]^ effectively improving the toughness of PUPAs. These results demonstrate that PUPA600‐1.5 has excellent mechanical properties.

**Figure 3:**
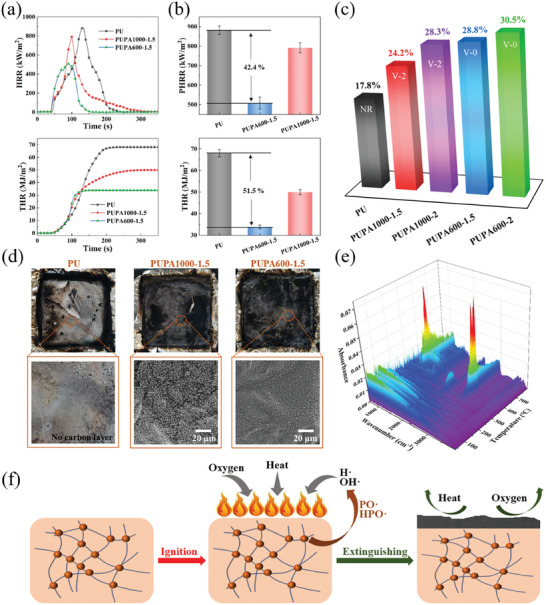
Flame‐retardant characterization of PUPAs. a) HRR and THR of PU, PUPA1000‐1.5, and PUPA600‐1.5 as function of time. b) PUPA600‐1.5 demonstrates the best flame retardancy with the lowest PHRR and THR values. c) LOI and flame‐retardant rating of PUPAs. d) Digital photos and SEM images of combustion residues after cone calorimeter test. e) TG–IR of PUPA600‐1.5. f) Schematic of possible flame‐retardant mechanism of PUPAs.

To evaluate the possibility of fabricating PUPAs into intrinsic flame‐retardant TENGs, the flame retardancy of PU, PUPA1000‐1.5, and PUPA600‐1.5 was tested using a cone calorimeter. The total heat release (THR) and peak heat release rate (PHRR) of the polymer indicate its ability to generate heat and the maximum heat release rate during combustion. The PHRR and THR values (508 kW/m^2^ and 33 MJ m^−2^) of PUPA600‐1.5 synthesized using PA–OH were 42.4% and 51.5% lower than those of the ordinary PU counterpart, respectively (**Figure** **3**a,b). Therefore, the synthesized PUPA can significantly decelerate the ignition and combustion process. Compared to the low limiting oxygen index (LOI = 17.8%) and flame‐retardant rating (NR) of the ordinary PU, PUPA600‐1.5 exhibited a significantly higher LOI of 28.8%, reaching the UL‐94 V0 level (Figure 3c; Table S4, Supporting Information), which is consistent with the cone calorimeter results.

Phosphoric acid is a bio‐based raw material that widely exists in plant seeds. PA–OH is prepared from phosphoric acid and introduced into polyurethane to obtain PUPA. The phosphorus‐containing segment of PUPA thermally decomposes and generates phosphonic acid upon exposure to flames. This can promote the dehydration and carbonization of PUPA, forming a carbon layer on the surface that blocks the contact between the polymer inside the carbon layer and oxygen, thus limiting the heat transfer to the interior (Figure 3d; Figure S7, Supporting Information). The phosphorus‐containing segment will simultaneously generate gases such as phosphorus oxides (Figure 3e; Figure S8, Supporting Information) when thermally decomposed. The further generated PO· and HPO· radicals can capture H· and OH· radicals released during polymer combustion, thereby blocking the chain reaction of combustion and decelerating the development of fire.^[^ ^17^ ^]^ The possible flame‐retardant mechanism of PUPA is shown in Figure 3f, the synergistic effect of which guarantees the safety of TENGs.

**Figure 4:**
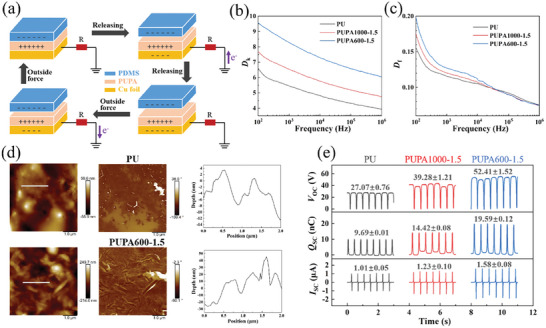
Working mechanism and electrical output performance of PUPA‐TENG. a) Schematic of operational mechanism of PUPA–TENG with layered structure. b) Dielectric constant (*D*_k_) and c) dissipation factor (*D*_f_) of PUPAs. d) Tapping‐mode atomic force microscopy (AFM) phase images of the surface of PUPAs and phase profiles of randomly selected scan paths (2 µm). e) Electrical output performance (*V*_oc_, *Q*_sc_, and *I*_sc_) of PUPA–TENGs with an operation frequency of 3 Hz.

Highly negative triboelectric materials, such as PDMS and PTFE, usually have low dielectric constants, and vice versa.^[^ ^18^ ^]^ A PDMS film with a dielectric constant of 2.2–2.75^[^ ^19^ ^]^ is used as a triboelectric material to construct a single‐electrode mode TENG, and the prepared PUPA film is used as the opposite positive triboelectric material. The schematic diagram and working mechanism of the PUPA‐TENG structure are shown in **Figure** **4**a. Driven by the physical contact between the PDMS and PUPA, they generate equivalent charges with opposite polarities. A potential difference between the sample and ground is generated upon separation of the PDMS and PUPA, and electrons flow from the ground to the electrode through an external circuit, thus generating electrical signals. In addition, when the PDMS layer is sufficiently distant from the PUPA layer, the induced negative charge on the electrode completely balances the positive charge on the PUPA, stopping the flow of electrons. Similarly, when the PDMS layer moves toward the PUPA layer once again, electrons will flow from the electrode to the ground, generating an opposite electrical signal; therefore, an alternating current is generated in such a circuit.

The dielectric properties of triboelectric materials are highly dependent on the polarity of their molecular structure.^[^ ^20^ ^]^ The dielectric constant of PUPAs significantly decreases as the frequency increases from 10^2^ to 10^6^ Hz (Figure 4b,c). At low frequencies, the polymer chain has sufficient time to respond, enabling the structure with the opposite polarity to react to the electric field. At high frequencies, the electric field rapidly changes, thus the molecular chain movement significantly lags, resulting in a low polarization and ultimately a lower dielectric constant.^[^ ^21^ ^]^ Conversely, the molecular chain of PUPA is more easily polarized owing to the introduction of PA–OH.^[^ ^21^ ^]^ Therefore, the dielectric constant of PUPA600‐1.5 (*D*_k_ = 9.57) is significantly higher than that of the traditional PU counterpart (*D*_k_ = 6.62). In addition to the dielectric constant, the surface roughness of the triboelectric layer is a critical factor for improving the electrical output performance of TENGs.^[^ ^22^ ^]^ The surface roughness of PUPAs was investigated using atomic force microscopy (AFM). The depth variation obtained from the AFM imaging of the tapping mode is an indicator of the surface roughness.^[^ ^23^ ^]^ Micro‐scale bulges formed on the surface of the PUPAs (Figure 4d), which can effectively increase the surface roughness and contact area between the triboelectric layers. Correspondingly, when PUPA600‐1.5 had a certain degree of surface roughness and a high dielectric constant, the open‐circuit voltage (*V*_oc_), short‐circuit charge (*Q*_sc_), and short‐circuit current (*I*_sc_) of PUPA‐TENG (52.4 V, 19.6 nC, and 1.6 µA, respectively) were significantly higher than those of PU–TENG composed of PU (*V*_oc_ = 27.1 V, *Q*_sc_ = 9.7 nC, *I*_sc_ = 1.0 µA) (Figure 4e). These results suggested that the PUPAs are promising triboelectric materials that can be used for fabricating self‐powered warning devices.

**Figure 5:**
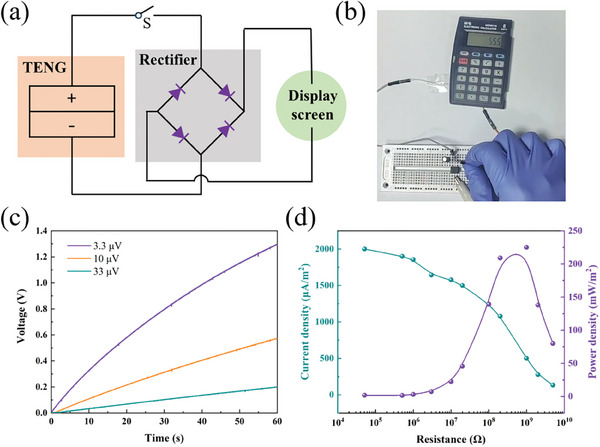
Electrical output performance of PUPA‐TENG. a) Circuit diagram of warning device. b) PUPA‐TENG was impacted by external forces and electrical signals were output to the display screen. c) Variations in voltage with charging time for different capacitors. d) Variations in current density and power density with external resistance.

Owing to its excellent flame‐retardancy and electrical output properties, PUPA600‐1.5 was selected as the triboelectric layer of PUPA‐TENG to explore its practical applications. As shown in **Figure** **5**a,b, at a working frequency of 3 Hz, the electrical signal generated by tapping PUPA‐TENG was easily transmitted to the display screen via wires and rectifiers. The charging efficiency of PUPA‐TENG was evaluated by using charging capacitors with different capacities (Figure 5c). After charging for 1 min, the capacitor voltage of 3.3 µV reached 1.25 V. In addition, the current density and power density of PUPA‐TENG were calculated, indicating that the power density reached a maximum value of 225 mW m^−2^ when the load resistance was 1 G Ω (Figure 5d).

Compared to the conventional TENGs limited by flammability and poor elasticity, PUPA600‐1.5 exhibited excellent intrinsic flame retardancy and elasticity characteristics as a new type of triboelectric material, indicating its feasibility for use in the protection of personnel in potential fire‐risk environments (Movie S1, Supporting Information). To verify the electrical output effect of PUPA‐TENG in practical applications, it was connected to an external display screen via wires and rectifiers to form a conductive path. When the mechanical signals were monitored by PUPA‐TENG, the display screen successfully lit up (Movie S2, Supporting Information). These results confirm the excellent flame‐retardancy, toughness, and electrical output performance of the bio‐based PUPA‐TENG without modifications or additives, and its significant potential for use in various fire‐risk applications such as personal protection and firefighting.

## 3 Conclusion

A series of low‐cost intrinsic flame‐retardant bio‐based polyurethanes (PUAPs) were prepared using a simple one‐pot polycondensation method. Among them, PUPA600‐1.5 demonstrated a fracture toughness of up to 34.71 MJ m^−3^, which was 45% higher than that of its typical PU counterpart. In addition, the limiting oxygen index of PUPA600‐1.5 was high (28.8%), the flame‐retardant rating reached the UL94 V‐0 level, and the dielectric constant was high (9.57), which was 50% higher than that of its typical PU counterpart. The bio‐based triboelectric material with the aforementioned properties is unprecedented. The bio‐based PUPA‐TENG prepared by using PUPA600‐1.5 demonstrated an excellent electrical output performance (*V*_oc_ = 52.4 V), indicating its promising potential for use in personal protective equipment in potentially fire‐risk environments, as well as in fire‐resistant energy collectors and rescue systems in extreme environments such as firefighting.

## 4 Experimental Section

### Materials

Polytetramethylene ether glycol (PTMEG, *M*_n_ = 1000 and *M*_n_ = 600) were provide by Macklin Reagent CO., Ltd. Isophorone diisocyanate (IPDI, 99%), Phytic acid solution (PA, 70% in water), Diglycerol (DG, 80%) and N,N‐dimethylformamide (DMF) were all provide by Aladdin Reagent CO., Ltd. Ditin butyl dilaurate (DBTDL) were provide by Beijing Zhengheng Chemical Co., Ltd.

### Synthesis of PA–OH

PA (13.2 g, 14 mmol) and DG (8.3 g, 40 mmol) were mixed in a three‐neck flask, reacted at 130 °C for 2 h, and placed in an 80 °C vacuum oven for 4 h to remove water, resulting in a fully bio‐based phytic acid polyol (PA–OH), as shown in Figure 1a and Figure S1 (Supporting Information). The possible molecular structure of PA–OH was determined using liquid‐phase ^13^C and ^31^P nuclear magnetic resonance spectroscopy. As shown in Figure S2 (Supporting Information), the adsorption band at 38.52 ppm in the ^13^C NMR spectrum corresponds to the CH_3_ group of DMSO‐d_6_. The signal peaks at 72.99, 75.22, and 76.37 ppm in the ^13^C NMR spectrum of PA corresponded to the CH group (C1–C6) of the inositol ring. Compared with the PA spectrum, the PA–OH spectrum demonstrated a new signal at 61.22 ppm corresponding to the CH_2_ group (C7–C12), and a new signal at 63.30 ppm corresponding to the CH_2_ group (C13–C24). Furthermore, the significant signal peak at the ^31^P position shifted from −1.99 to −1.00 ppm owing to the formation of covalent phosphate bonds,^[^ ^24^ ^]^ which confirms the preparation of PA–OH.

### Synthesis of PUPAs

A series of PUPAs were prepared using the standard stepwise polymerization method (Figure 1a; Table S1, (Supporting Information), and labeled according to the molecular weight of PTMEG and the weight of PA–OH. For example, the combination of PTMEG (*M*_n_ = 600) and PA–OH (1.5 g) was defined as ″PUPA600‐1.5″. Table S1 (Supporting Information) summarizes the composition of PUPAs. As an example, the PUPA600‐1.5 was prepared as the following procedure: PTMEG (*M*_n_ = 600, 3.48 g, 5.80 mmol), IPDI (2.22 g, 10 mmol) were separately weighed and dissolved in 5 mL DMF, reacted at 60 °C under nitrogen atmosphere for 60 min. Subsequently, PA–OH (1.5 g, 7.05 mmol‐OH) dissolved in 3 mL of DMF was added, and the mixture was further reacted under a nitrogen atmosphere at 60 °C for 120 min, resulting in a light yellow viscous liquid; the above product was poured into a polytetrafluoroethylene mold, placed in a vacuum oven at 60 °C defoaming for 2 h, and then cured in air‐blowing oven at 80 °C for 24 h to obtain the PUPA600‐1.5. Infrared spectroscopy and energy dispersive spectrometer (EDS) tests were performed on the raw materials and products to characterize whether the PUPAs were successfully synthesized. As shown in Figure S3 (Supporting Information), both characteristic peaks of ─N═C═O at 2250 cm^−1^ and ─OH at 3500 cm^−1^ completely disappeared for the PUPAs, meanwhile the bending vibration peak of P─O─C appeared at 490 cm^−1^, indicating that the reaction between isophorone diisocyanate (IPDI), polytetramethylene ether glycol (PTMEG), and PA–OH was completed. A phosphorus element was detected in the PUPAs instead of PU (Figure S4 and Table S2, Supporting Information), thus indicating the preparation of PUPA.

## Conflict of Interest

The authors declare no conflict of interest.

## Supporting information



Supporting Information

Supplemental Movie 1

Supplemental Movie 2

## Data Availability

The data that support the findings of this study are available from the corresponding author upon reasonable request.
