# Supplementary material for: Low‐Cost Intrinsic Flame‐Retardant Bio‐Based High Performance Polyurethane and its Application in Triboelectric Nanogenerators
Source: Adv Sci (Weinh). 2024 Dec 30;12(8):2412258. doi: 10.1002/advs.202412258 (PMC11848539; doi:10.1002/advs.202412258)
Supplement: Supplementary file 1 — Supporting Information [file ADVS-12-2412258-s003.docx]

**Supporting Information**

**Low-cost intrinsic flame-retardant bio-based polyurethane construction Li-ion power battery thermal runaway self-powered warning device**

Xiaoyu Zhang^1†^, Xixian Yan^1†^, Fanglei Zeng^1†^, Hao Zhang^1^, Peiyao Li^1^, Haiyang Zhang^2^, Ning Li^1,^*, Qingbao Guan^2,^*, and Zhengwei You^2,^*

1. Jiangsu Collaborative Innovation Center for Photovoltaic Science and Engineering, Jiangsu Province Cultivation Base for State Key Laboratory of Photovoltaic Science and Technology; Jiangsu Province Key Laboratory of Environmentally Friendly Polymer Materials, School of Materials Science and Engineering, Changzhou University, Changzhou 213164, P. R. China.

2. State Key Laboratory for Modification of Chemical Fibers and Polymer Materials, College of Materials Science and Engineering, Institute of Functional Materials, Research Base of Textile Materials for Flexible Electronics and Biomedical Applications (China Textile Engineering Society), Shanghai Engineering Research Center of Nano Biomaterials and Regenerative Medicine, Donghua University; Shanghai 201620, P. R. China.

*Correspondence: czlin20201116@163.com (N.L.); qbguan@dhu.edu.cn (Q.G.); zyou@dhu.edu.cn (Z.Y.)

† These authors contributed to this work equally.

**Content：**

**Experimental Section: Characterizations**

**Figure S1. Synthetic schemes of PUPAs.**

**Figure S2. NMR spectra of PA and PA-OH.**

**Figure S3. FTIR spectra of raw materials and products.**

**Figure S4. EDS spectra of PUPAs.**

**Figure S5. Gel content of PUPAs.**

**Figure S6. Anti-puncture diagram of PUPAs.**

**Figure S7. EDS spectra of carbon layer.**

**Figure S8. FTIR spectrum of volatile pyrolysis products of PUPA600-1.5.**

**Table S1. Raw material ratio of a series of PUPAs.**

**Table S2. The content of different elements in the products measured by EDS.**

**Table S3. Thermal stability data of PUPAs.**

**Table S4. Flame retardant data of PUPAs.**

**Experimental Section**

*Characterizations*: The differential scanning calorimeter (DSC) tests were carried out using a Q2000 (TA). In this regard, 10 ~ 15 mg of each sample was precisely weighted and sealed in aluminum pans. The tests were performed in the temperature range of -30 °C to 80 °C with a heating rate of 10 °C∙min^−1^ under a nitrogen atmosphere.

Thermogravimetric analysis (TGA) tests were performed on a STA6000 synchronous thermal analysis analyzer (PerkinElmer). The adequate amount of each sample was placed in the pan and tested in the temperature range of 30 °C to 800 °C under a nitrogen atmosphere, while the heating rate was set at 20 °C∙min^−1^ for all experiments.

Universal material testing machine (WT-10) was used for mechanical property test, the tensile rate is 50 mm/min^-1^, and the test temperature is 25 ℃.

WATERS Breeze GPC (Waters Company, USA) was used to test, utilize Styragel HR4E chromatographic column, N,N-dimethylformamide (DMF) used as the test solvent, flow rate of 1.0 mL∙min^-1^.

Characterize the chemical structure of raw materials and products was used fourier transform infrared spectroscopy (Nicolt A vatar370). With 16 scanning times, a resolution of 4 cm^-1^ and a scanning range of 4000 cm^-1^ ~ 400 cm^-1^.

The ^13^C and ^31^P liquid state nuclear magnetic resonance (NMR) spectra were recorded on the Bruker Avance III 400 MHz spectrometer (Bruker BioSpin GmbH, Germany) using DMSO-d_6_ as a deuterated solvent.

The surface morphology of the prepared PUPAs was investigated using scanning electron microscopy (JSM-IT100, Japan) and energy dispersive spectroscopy (EDS) operating at 5 kV. The samples were fixed on the sample holder using conductive adhesive tape and then gold coated for 60 s before testing.

The basic chemical thermal values were measured by cone calorimeter (FTT0007). The sample with the size of 100 mm × 100 mm × 3 mm.

The dielectric constant (*D*_k_) and dissipation factor (*D*_f_) were recorded with a broadband dielectric impedance spectrometer (Agilent4294A). Frequency multiplexing was adjusted at 10^2^ ~ 10^6^ Hz and a test temperature of 25 °C. The samples were hot pressed into sheets, cut into wafers with a diameter of 20 mm and a thickness of less than 2 mm, and laminated with copper foil on both sides.

Surface morphology of specimens were investigated using Atomic force microscopy (Bruker Dimension Icon) with an aluminum reflex-coated silicon cantilever probe (Tap AC160TS-R3). The AFM tip has a half cone angle of 10°, a radius of 7 nm, and a resonance frequency of 300 kHz. The nominal force constant was set at 26 N·m^−1^ and the probe's actual spring constant was precisely calibrated before each test.

The output voltage, current and charge of the triboelectric nanogenerator (TENG) were evaluated using a Keithley DMM7510 system electrometer. The samples with the size of (30 ± 1) mm × (30 ± 1) mm × (0.2 ± 0.05) mm were measured at an operation frequency of 3 Hz.


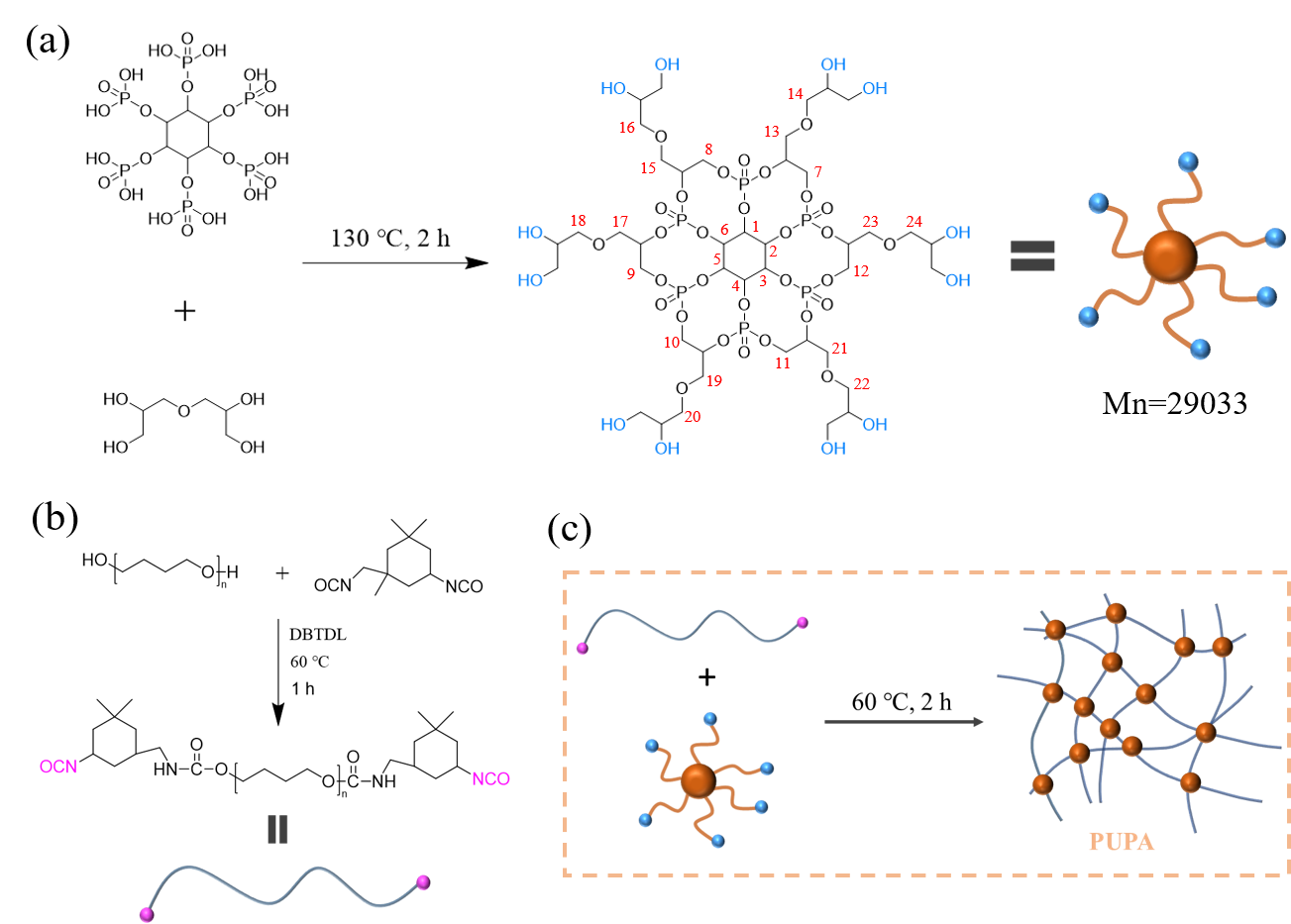


**Figure S1. Synthetic schemes of PUPAs.** (a) Synthesis of PA-OH based on phytic acid and diglycerol. (b) Synthesis of polyurethane prepolymer. (c) Synthesis of PUPA.


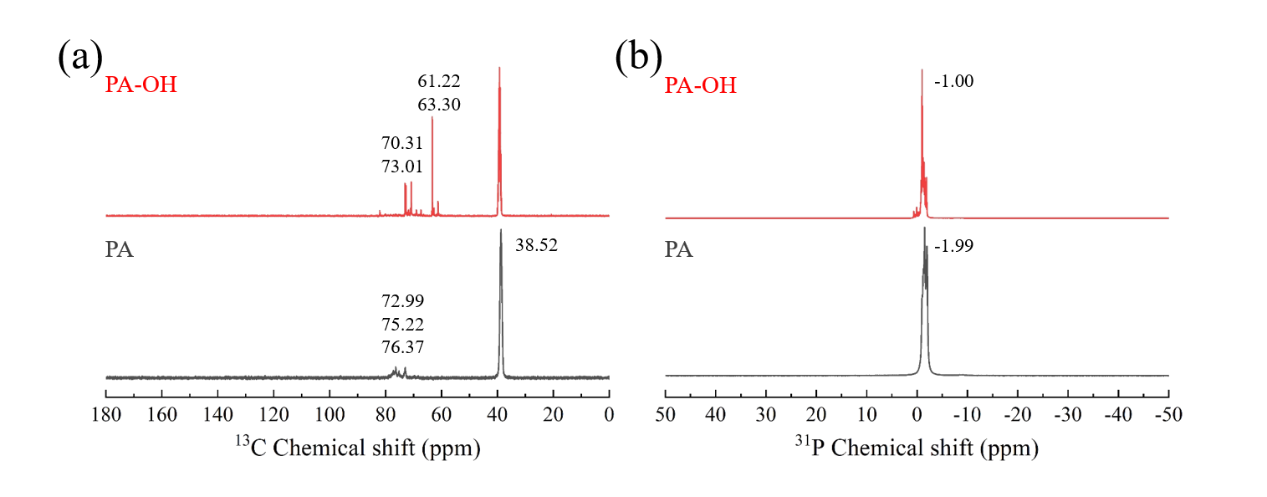


**Figure S2.** **NMR spectra of PA and PA-OH.**


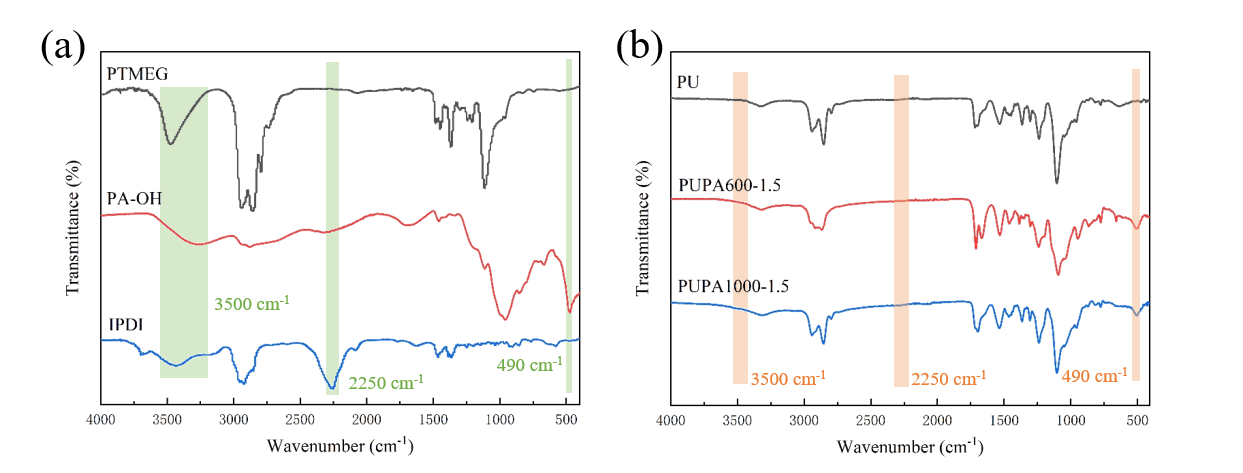


**Figure S3.** **FTIR spectra of raw materials and products.**


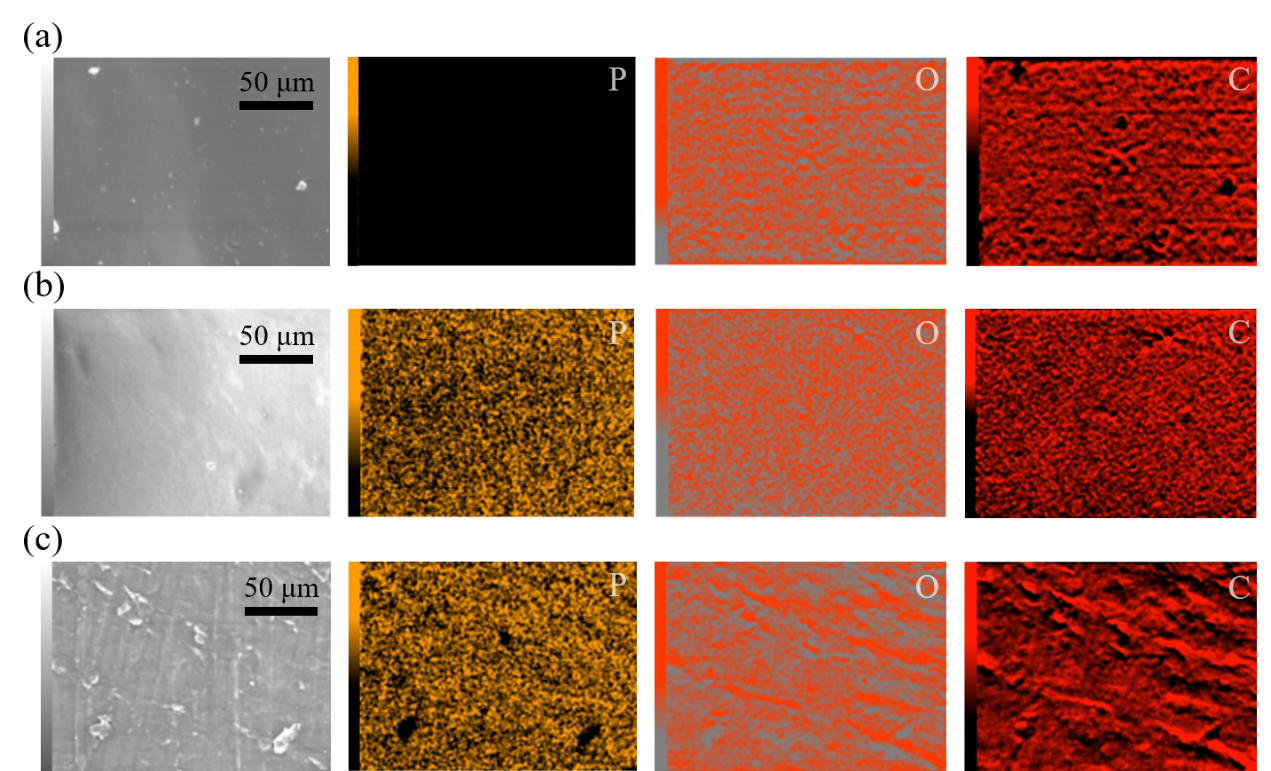


**Figure S4.** **EDS spectra of PUPAs.** (a) PU, (b) PUPA1000-1.5, (c) PUPA6001.5


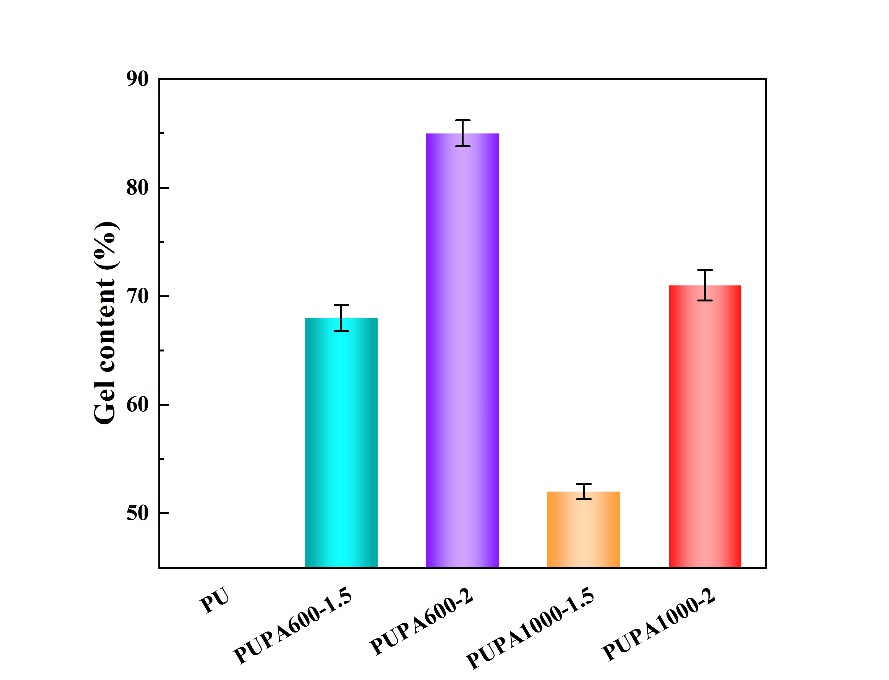


**Figure S5.** **Gel content of PUPAs.**


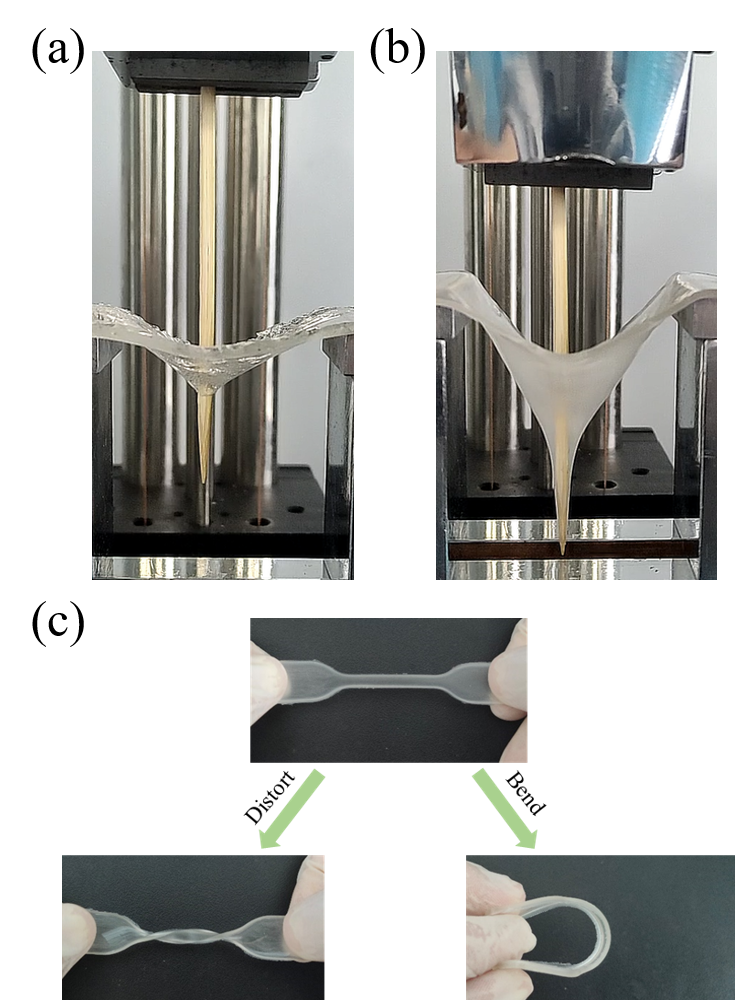


**Figure S6. Anti-puncture and elasticity of PUPAs.** (a) PU, (b) PUPA1000-1.5 and (c) deformation of PUPA600-1.5


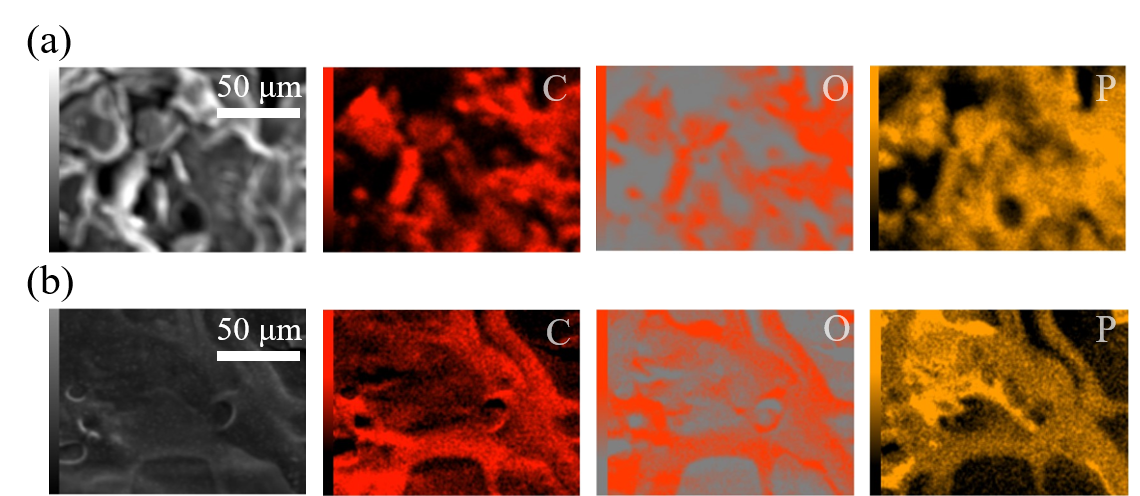


**Figure S7. EDS spectra of carbon layer.** (a) PUPA1000-1.5, (b) PUPA600-1.5


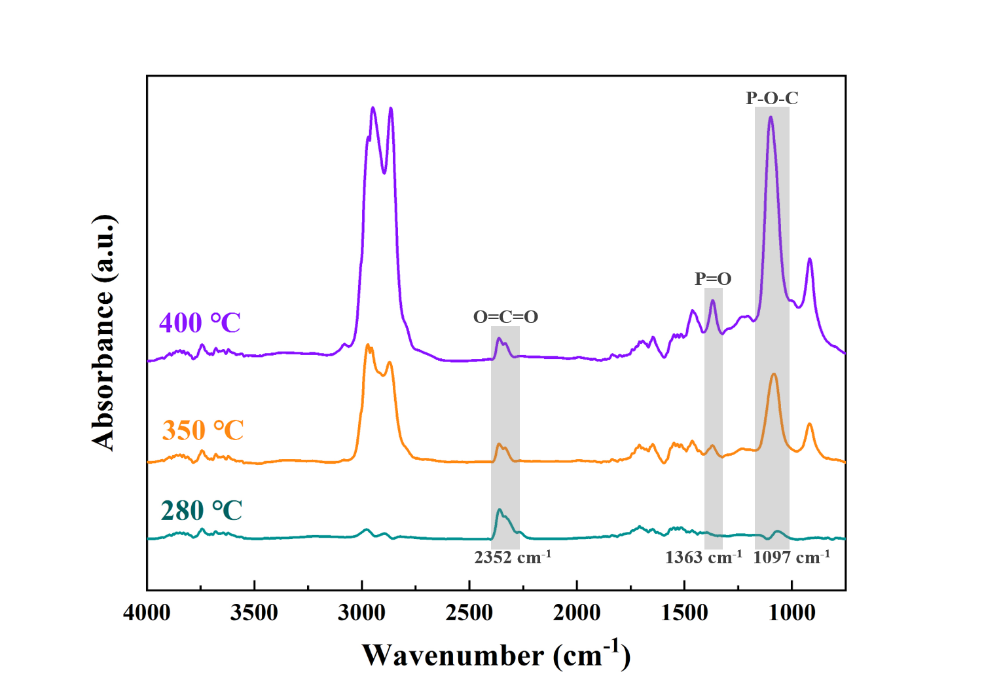


**Figure S8.** **FTIR spectrum of volatile pyrolysis products of PUPA600-1.5.**

**Table S1.** **Raw material ratio of a series of PUPAs**

| Samples | PTMEG600 | | PTMEG1000 | | IPDI | | PA-OH | |
| --- | --- | --- | --- | --- | --- | --- | --- | --- |
|  | Mass (g) | mmol | Mass (g) | mmol | Mass (g) | mmol | Mass (g) | -OH mmol |
| PU | / | / | 9.00 | 9.00 | 2.22 | 10.00 | / | / |
| PUPA1000-1.5 | / | / | 5.80 | 5.80 | 2.22 | 10.00 | 1.50 | 7.05 |
| PUPA1000-2 | / | / | 4.70 | 4.70 | 2.22 | 10.00 | 2.00 | 9.40 |
| PUPA600-1.5 | 3.48 | 5.80 | / | / | 2.22 | 10.00 | 1.50 | 7.05 |
| PUPA600-2 | 2.82 | 4.70 | / | / | 2.22 | 10.00 | 2.00 | 9.40 |

**Table S2.** **The content of different elements in the products measured by EDS**

| Samples | C content | | O content | | P content | | N content | |
| --- | --- | --- | --- | --- | --- | --- | --- | --- |
|  | Mass% | Atom% | Mass% | Atom% | Mass% | Atom% | Mass% | Atom% |
| PU | 66.39 | 71.92 | 32.70 | 27.21 | 0 | 0 | 0.91 | 0.87 |
| PUPA1000-1.5 | 64.32 | 72.1 | 31.81 | 26.96 | 2.90 | 1.26 | 0.97 | 0.94 |
| PUPA600-1.5 | 61.08 | 68.88 | 33.03 | 27.96 | 4.79 | 2.10 | 1.10 | 1.06 |

**Table S3.** **Thermal stability data of PUPAs**

| **Samples** | ***T*_d5%_ (℃)** | **Char yield (%)** |
| --- | --- | --- |
| PU | 325 | 0 |
| PUPA1000-1.5 | 280 | 5.52 |
| PUPA1000-2 | 268 | 7.96 |
| PUPA600-1.5 | 223 | 7.92 |
| PUPA600-2 | 207 | 9.87 |

**Table S4. Flame retardant data of PUPAs**

| Samples | LOI (%) | UL-94 rating | t_1_/t_2_ (s) | dripping | Cotton ignition |
| --- | --- | --- | --- | --- | --- |
| PU | 17.8 ± 0.3 | NR | burn out | Yes | Yes |
| PUPA1000-1.5 | 26.2 ± 0.1 | V-2 | 6.5 ± 0.5/7.1 ± 0.7 | Yes | Yes |
| PUPA1000-2 | 28.3 ± 0.2 | V-0 | 2.0 ± 0.6/3.2 ± 0.4 | Yes | No |
| PUPA600-1.5 | 28.8 ± 0.2 | V-0 | 1.2 ± 0.2/1.8 ± 0.2 | Yes | No |
| PUPA600-2 | 30.5 ± 0.2 | V-0 | 1.0 ± 0.2/1.4 ± 0.3 | Yes | No |
